# The effects of tyrosol on yeasts: an overview of current knowledge

**DOI:** 10.1007/s00253-025-13595-y

**Published:** 2025-09-12

**Authors:** Renátó Kovács, Ágnes Jakab

**Affiliations:** 1 Department of Medical Microbiology, Faculty of Medicine, University of Debrecen, Nagyerdei Krt. 98., 4032 Debrecen, Hungary; 2 Medical Microbiology, Clinical Centre, University of Debrecen, Nagyerdei Krt. 98., 4032 Debrecen, Hungary

**Keywords:** Tyrosol, *Candida*, *Saccharomyces*, Quorum sensing, Biotechnology, Antifungal therapy, Biofilm

## Abstract

**Abstract:**

Quorum sensing is a cell density-dependent microbial communication form, which can regulate several microbial properties, including virulence, biofilm formation and cell–cell competence. The phenomenon of fungal quorum sensing was first uncovered nearly 25 years ago, following the identification of farnesol and tyrosol as two key signalling molecules. Although the major roles of these regulatory molecules were elucidated, several questions primarily regarding tyrosol-mediated effects remain to be addressed, particularly with regard to molecular events influenced by tyrosol. Based on available literature data, tyrosol possesses potential antifungal activity, especially at supraphysiological concentrations. Moreover, its simultaneous usage with traditional antifungals shows potent synergistic activity against planktonic and sessile *Candida* cells, including both *Candida albicans* and certain non-albicans species. Currently, the deep molecular tyrosol-based investigations are still in their infancy compared with farnesol research. However, several promising findings were published in the past 10 years in terms of the potential usage of this compound as an alternative therapeutic treatment. Hence, this mini review summarizes the major functions of tyrosol as a signaling regulator compound in *Candida* morphogenesis. Furthermore, we discussed the most promising tyrosol-based in vitro data, which may be a foundation for the future development of in vivo models and ultimately innovative therapeutic strategies against fungal infections.

**Key points:**

• *Tyrosol is a major quorum-sensing molecule in Candida species, promoting yeast-to-hyphae transition and biofilm formation*

• *Tyrosol has been shown to potentiate the efficacy of conventional antifungal agents, representing a promising adjunctive strategy for the treatment of fungal biofilms*

• *At supraphysiological concentrations, tyrosol induces oxidative stress, negatively influences the intracellular metal homeostasis and alters the fungal metabolism*

## Introduction

Quorum sensing is a population density-dependent cell signaling mechanism in microbes, which plays a pivotal role in regulating of virulence properties and biofilm development (Mukherjee and Bassler 2019; Tian et al. 2021). Quorum-sensing-related pathways are mediated by low-molecular-weight secreted molecules with a variety of biological activities, which are not essential in central metabolism (Mukherjee and Bassler 2019; Tian et al. 2021). Quorum sensing was a relatively unknown phenomenon in fungal species until Hornby et al. (2001) described the effect of the farnesol (3,7,11-trimethyl-2,6,10-dodecatriene-1-ol) on *Candida albicans* morphogenesis, where it inhibits the morphological switching from yeast to hyphae at high cell densities as well as blocks germ tube formation. In addition to farnesol, tyrosol was the second quorum-sensing molecule discovered in fungal species, even though their role as autoinducers in *C. albicans* had already been suggested in the 1970 s (Lingappa et al. 1969).

Tyrosol (2-[4-hydroxyphenyl] ethanol), a tyrosine-derived molecule, is a key phenolic compound found in olive oil and is recognized for its potent antioxidant properties (Moreno et al. 2003). Phenolic compounds are a class of secondary metabolites widely found in plants, characterized by one or more benzene rings bearing one or more hydroxyl groups. Tyrosol as a typical phenolic compound, with a chemical structure consisting of a benzene ring, a hydroxyl group, and a hydroxyethyl side chain. The phenolic hydroxyl group is critical for both its antioxidant properties and antimicrobial activity. The hydroxyethyl side chain allows tyrosol to integrate into plasma membranes and modulate membrane-related processes. As a major chemical intermediate, it has wide applications in chemistry, pharmaceuticals and agriculture (Wang et al. 2025a, b).

Chen et al. (2004) later identified tyrosol as a quorum-sensing molecule in *C. albicans*. Based on their initial experiments, its growth stimulatory activity was described in *C. albicans* (Chen et al. 2004). Tyrosol is continuously released into the growth medium during *Candida* growth and can specifically abolish the lag phase at a concentration of ≥ 10 μM, with no effect observed in the exponential phase. Moreover, it significantly accelerated the morphological conversion of *C. albicans* from yeast to filamentous forms (Chen et al. 2004). Previous studies have demonstrated that the effect of tyrosol on the regulation of morphogenesis in *C. albicans* is secondary to farnesol and is therefore considered a minor quorum-sensing molecule whose influence is detectable only when farnesol is either limited or absent in the environment (Nickerson et al. 2006). The observed morphological and growth-related changes are associated with a number of genes encoding the DNA-replication machinery and cell–cell control proteins, which are critical for cell division (Chen et al. 2004).

Based on several in vitro and in vivo studies, the usage of quorum-sensing molecules, including tyrosol, primarily at supraphysiological concentrations, may have adverse effects on cell–cell communication processes in fungal planktonic cells and biofilms (Katragkou et al. 2015; Cordeiro et al. 2013; Monteiro et al. 2015; Cordeiro et al. 2015; Arias et al. 2016; Bozó et al. 2016; Kovács et al. 2016; Kovács et al. 2017; Monteiro et al. 2017a, b; Nagy et al. 2020a; Nagy et al. 2020b; Hacioglu et al. 2024; Jakab et al. 2024). In this mini-review, we provide an overview of current knowledge of the effects of tyrosol on yeast cells, its industrial importance and its potential future perspectives as an alternative antifungal therapy.

### Effects of tyrosol on planktonic forms of yeasts

**Fig. 1:**
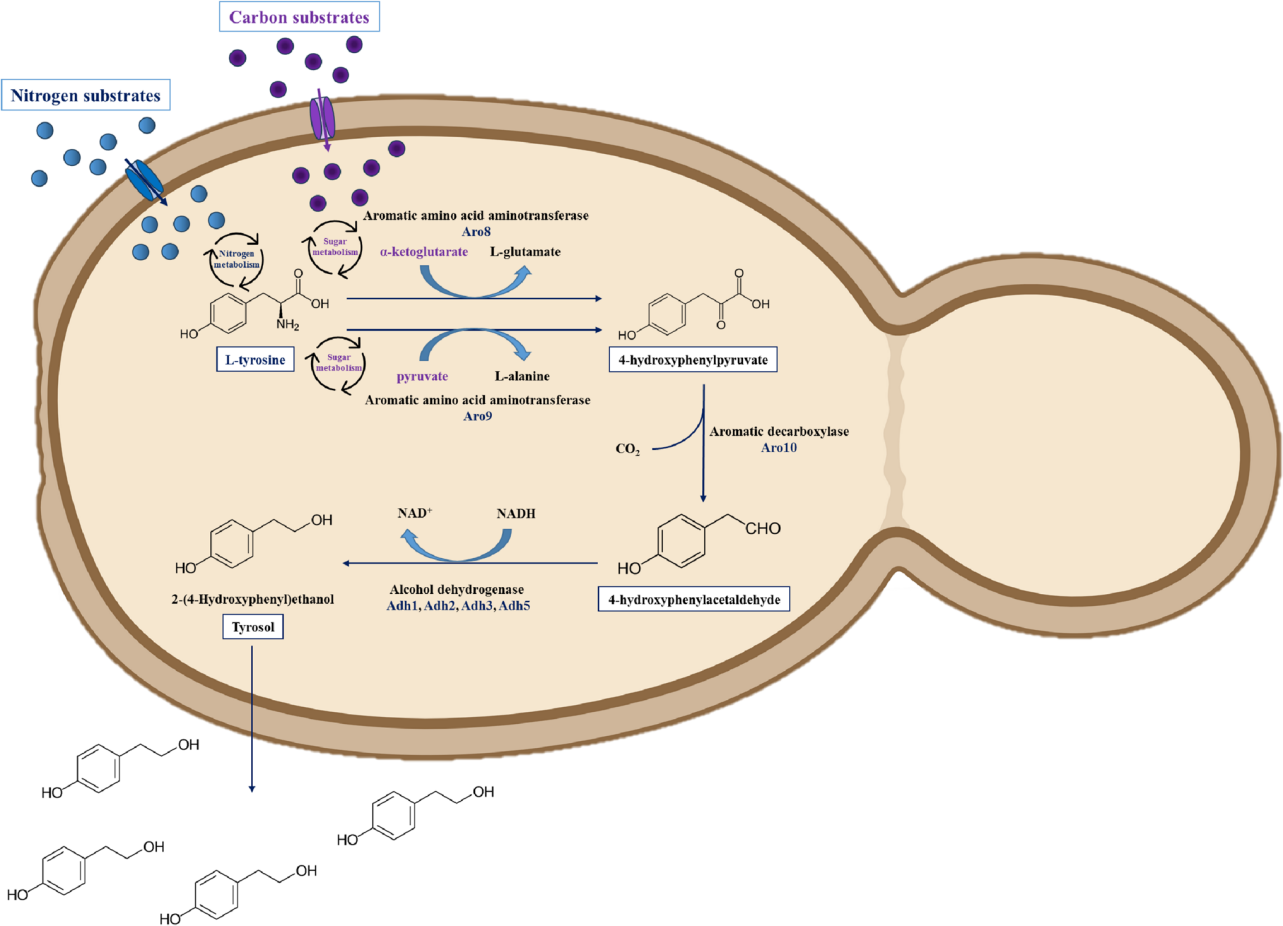
Scheme of biosynthesis of tyrosol in *Candida albicans*. Created in BioRender. J, A. (2025) https://BioRender.com/oyblz4z

The biosynthesis of tyrosol in *Candida* cells primarily occurs through the catabolism of tyrosine via the Ehrlich pathway (Ghosh et al. 2008; Avbelj et al. 2016) (Fig. 1). Briefly, L-tyrosine from the medium or shikimate pathway is transaminated to 4-hydroxyphenylpyruvate, catalysed by Aro8 and Aro9. Afterwards, it is decarboxylated to 4-hydroxyphenylacetaldehyde, mediated by Aro10, and finally reduced to tyrosol by alcohol dehydrogenase enzymes (Adh1, Adh2, or Adh6) (Fig. 1). The production of aromatic alcohols, including tyrosol, is significantly influenced by growth conditions, oxygen contents, aromatic amino acids, ammonium salt availability, and pH (Ghosh et al. 2008; Avbelj et al. 2016). Based on previous studies, tyrosol production could increase by adding tyrosine or ammonium salts into the growth medium (Ghosh et al. 2008). Ghosh et al. (2008) reported that the transcription regulator Aro80p has a pivotal role in tyrosol production. Tyrosol synthesis-related genes, such as *ARO8*, *ARO9* and *ARO10*, showed typical pH-dependent expression. Based on their findings, *ARO8* and *ARO9* are alkaline up-regulated, while *ARO10* showed down-regulation under alkaline conditions. Further differences in *ARO* gene expression that *ARO8* showed a Rim101-independent (a pH response transcription factor) alteration, whereas *ARO9* expression was Rim101-dependent (Ghosh et al. 2008). Besides these genes, *DPP3* may also have a remarkable effect on tyrosol production, at least in a species-specific manner. In *C. lusitaniae*, the production of tyrosol was surprisingly altered in the *dpp3*Δ knockout mutant, which may be associated with altered macrophagic functions (Sabra et al. 2014).

Chen et al. (2004) reported the continuous accumulation of tyrosol into the extracellular area at a low concentration (~ 3 µmol/L) at ≥ 10^8^ cells/mL cellular density, showing that tyrosol is released into the medium continuously during the growth. Based on their findings, tyrosol is not a crucial growth factor because it could not be used as a carbon source; furthermore, it did not influence the rate of exponential growth (Chen et al. 2004). Interestingly, tyrosol, in addition to its concentration- and time-dependent activity, exhibited a marked temperature-dependent effect on the growth of *Candida* cells (Márton et al. 2023).

Cremer et al. (1999) found that multiple *Candida* species produce tyrosol, and that *C. tropicalis* expressed it in the highest quantities (ranging from 41.2 ± 1.2 to 48.6 ± 3.8 μM per 2.6 × 10^7^–2.7 × 10^7^ colony forming unit (CFU)/mL) followed by *C. albicans* (ranging from 21.0 ± 0.8 to 53.4 ± 1.7 μM per 1.6 × 10^7^–5.3 × 10^7^ CFU/mL), *C. glabrata* (ranging from 1.3 ± 0.2 to 3.3 ± 0.33 μM per 2.7 × 10^7^–5.5 × 10^7^ CFU/mL), and *C. parapsilosis* (ranging from 1.6 ± 0.3 to 3.0 ± 0.4 μM per 1.7 × 10^7^–2.3 × 10^7^ CFU/mL), suggesting a possible link with virulence. When tyrosol production was normalized to CFU, distinct species-specific differences became apparent: *C. tropicalis* and *C. albicans* displayed the highest yields (10⁻⁷–10⁻⁶ nmol/CFU), whereas *C. glabrata* and *C. parapsilosis* produced lower amounts (10⁻⁸–10⁻⁷ nmol/CFU). Besides *Candida* species, remarkable tyrosol production was also detected in *Saccharomyces cerevisiae* (72.9 ± 1.1 µM/L) (Soejima et al. 2012); however, it had no detectable effect in this species at least with regard to growth. Gori et al. (2011) also reported the presence of tyrosol in *Debaryomyces hansenii*, depending on cell density and environmental conditions. Based on gas chromatography–mass spectrometry analysis, *C. auris* also secreted tyrosol, indicating that tyrosol is a critical *C. auris* morphogenic metabolite under the given condition tested by Semreen et al. (2019).

Several studies have shown that externally added tyrosol at supraphysiological concentrations inhibits the growth of *Candida* cells. Within two hours of addition, 15 mM of tyrosol significantly inhibited the growth of *C. parapsilosis* (8.3 × 10^7^ ± 0.8 × 10^7^ and 4.5 × 10^7^ ± 0.4 × 10^7^ cells/mL for untreated control and tyrosol-exposed cells, respectively) and *C. auris* (5.6 × 10^7^ ± 1.2 × 10^7^ and 2.5 × 10^7^ ± 0.6 × 10^7^ cells/mL for control and tyrosol-treated cells, respectively). Nevertheless, 2-h-long tyrosol exposure (15 mM) did not significantly change the ratios of yeast cells and pseudohyphae in these non-albicans species (Jakab et al. 2019; Balla et al. 2023). In the case of *C. albicans*, do Vale et al. (2017) observed a significant decrease in the amount of hyphae and pseudophyphae in the presence of 1.25 mM tyrosol at 48 h compared with the untreated negative control. Surprisingly, a similar range of tyrosol concentrations (1 to 20 mM) did not significantly inhibit the growth of the *C. albicans* ATCC 10231 reference strain (Sebaa et al. 2019). Nevertheless, tyrosol at 25, 50, 100, and 200 mM reduced the number of germ tubes by 67%, 92%, 97%, and 97% in *C. albicans*, respectively (Monteiro et al. 2015). For the virulence factors examined, tyrosol exposure had no effect on the extracellular proteinase, phospholipase, and lipase activity, at least for non-albicans species (Jakab et al. 2019; Balla et al. 2023).

### Effects of tyrosol on biofilm development of yeasts

**Fig. 2:**
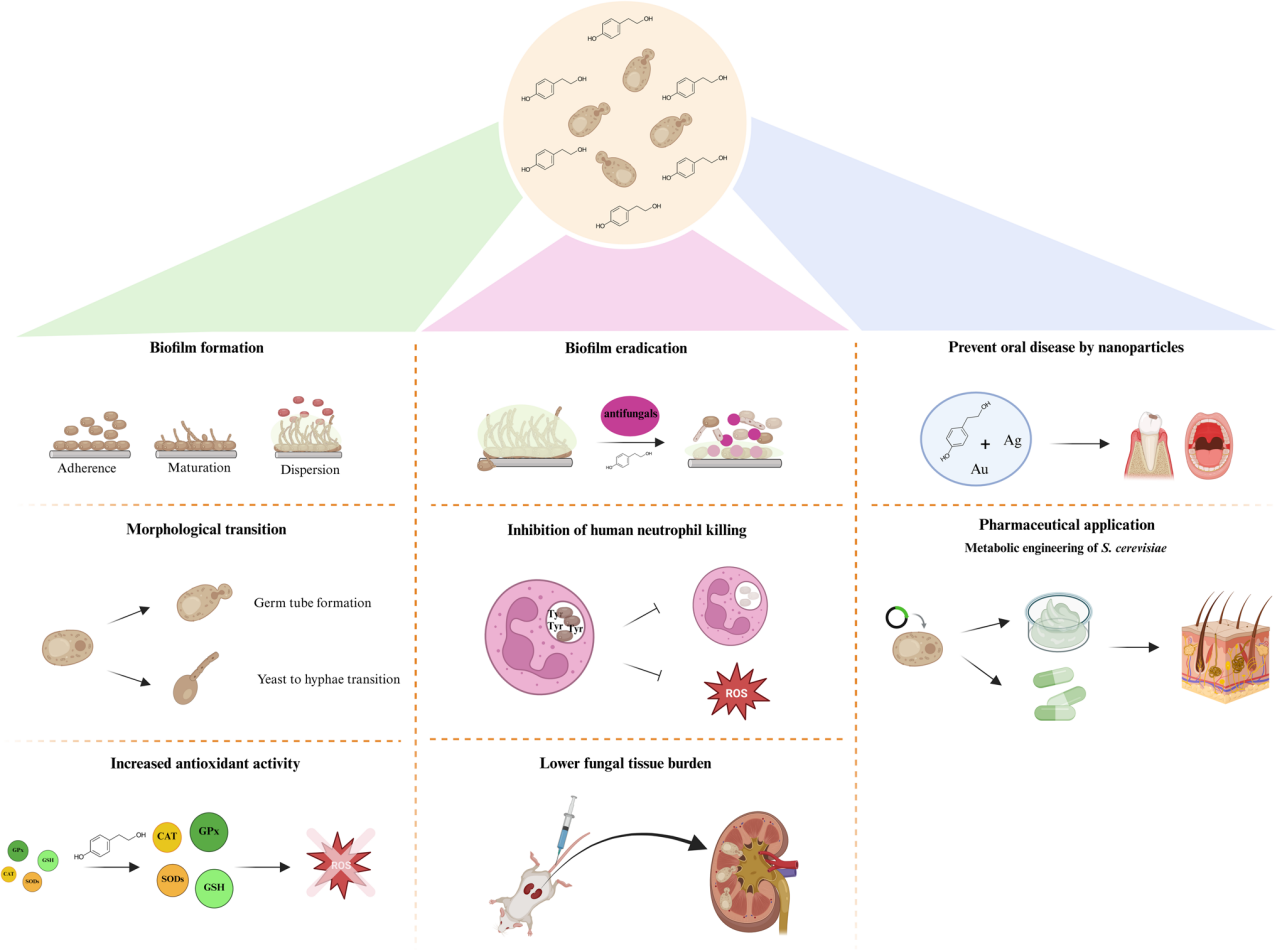
Effects and potential usage of tyrosol. Created in BioRender. J, A. (2025) https://BioRender.com/zgqi6w2

Tyrosol production plays a crucial role in fungal morphogenesis and biofilm development in various *Candida* species (Fig. 2). Alem et al. (2006) described that tyrosol induces hypha development throughout the initial stages of biofilm formation (1 to 6 h) and intermediate stages of biofilm development prior to some *C. albicans* cells being already committed to filamentous growth. It is noteworthy, that this biofilm-forming inducer effect has been recognised in certain non-albicans species, such as *C. auris*, to grow as yeast or pseudohyphae (Semreen et al. 2019). Results obtained from *C. albicans* biofilms showed its potentiating effect on biofilm development (Fig. 2). Tyrosol production was 9.6 ± 0.5 nmol/mg (dry weight) and 5.7 ± 1.1 nmol/mg (dry weight) for two-day-old biofilms and planktonic cells, respectively (Alem et al. 2006). Although *C. albicans* mutants lacking the Efg1 pathway, the Cph1 pathway, or both released tyrosol in a density-dependent manner and at levels comparable to the wild-type strain, tyrosol had no impact on the biofilm morphologies formed by the cph1/cph1, efg1/efg1, or the double mutant cph1/cph1 efg1/efg1 strains (Alem et al. 2006).

Exogenously added tyrosol at supraphysiological concentrations did not significantly influence the biofilm-forming properties of *C. parapsilosis* where the living fungal cells were comparable in the presence or absence of 15 mM tyrosol (4.9 × 10^5^ ± 1.1 × 10^5^ and 6.4 × 10^5^ ± 1.9 × 10^5^ living fungal cells at 24 h for control and tyrosol-treated sessile cells, respectively); nevertheless, metabolic activity of adhered cells was significantly and compensatory increased in the tyrosol treatment group (Jakab et al. 2019).

### Antifungal potential of tyrosol against yeasts

Recently, therapeutic approaches interfering with quorum sensing have become an attractive area in alternative treatment strategies (Rodrigues and Černáková 2020; Kovács and Majoros 2020). The reported inhibitory effect of quorum-sensing molecules (e.g., farnesol, tyrosol), especially at supraphysiological concentrations, suggests their potential use in monotherapy or in combination with traditional antifungal agents, providing novel opportunities in the management of potentially hard-to-treat fungal species, such as fluconazole-resistant *C. parapsilosis* or *C. auris* (Rodrigues and Černáková 2020; Kovács and Majoros 2020).

Based on previously published minimal inhibitory concentrations, tyrosol exerted at least 50% growth inhibition at concentrations of > 1 mM, 5 mM, 50 mM, and 90 mM for *C. parapsilosis, C. tropicalis*, *C. albicans*, and *C. glabrata*, respectively, which correspond to supraphysiological values (Cordeiro et al. 2015; Monteiro et al. 2015; Arias et al. 2016; do Vale et al. 2017; Kovács et al. 2017; Monteiro et al. 2017a, b). In in vivo studies, daily administration of 15 mM tyrosol resulted in at least a 0.5–1 log reduction in kidney fungal burden in immunocompromised mouse model (Jakab et al. 2019) (Fig. 2).

Regarding tyrosol-drug combinations, generally, an isolate-dependent synergistic activity can be seen for planktonic cells. Cordeiro et al. (2015) reported 55% (Fractional Inhibitory Concentration Index (FICI): 0.077 to 0.375), 90% (FICI: 0.046 to 0.5), and 90% (FICI: 0.0175 to 0.5) synergism for *C. albicans* and *C. tropicalis* for amphotericin B-tyrosol, itraconazole-tyrosol, and fluconazole-tyrosol combinations, respectively. Kovács et al. (2017) did not observe an inhibitory effect of tyrosol at concentrations lower than 1 mM; furthermore, the combinations with echinocandins were indifferent against planktonic *C. parapsilosis*. Jakab et al. (2019) reported an antagonistic interaction between tyrosol and fluconazole against *C. parapsilosis* planktonic cells (FICI: 4.125) (Jakab et al. 2019). In line with these findings, Noskova et al. (2023) showed that when tyrosol is added simultaneously with the other azole drug, clotrimazole, it is able to cancel its fungistatic activity and restore cell growth, further proving the antagonistic interactions between tyrosol and azoles (Fig. 2).

Sessile minimum inhibitory concentration (MIC) values to tyrosol were significantly higher compared with planktonic cells. Based on XTT (2,3-Bis-(2-Methoxy-4-Nitro-5-Sulfophenyl)−2*H*-Tetrazolium-5-Carboxanilide) assay-based susceptibility testing, the highest reductions in metabolic activity were observed at concentrations higher than 200 mM (Cordeiro et al. 2015; Monteiro et al. 2015). Concerning tyrosol-antifungal drug combinations, a concentration-dependent synergistic effect was generally reported. Shanmughapriya et al. (2014) showed that the combination of tyrosol with amphotericin B exhibited synergistic activity against *C. krusei* and *C. tropicalis* sessile communities, achieving a 90% reduction at 80 μM and 4 mg/L, respectively. *C. albicans* and *C. tropicalis* biofilm development decreased by at least 60% in metabolic activity following treatment with amphotericin B, fluconazole, and itraconazole in the presence of tyrosol. Interestingly, Jakab et al. (2019) observed a clear antagonistic interaction between tyrosol and fluconazole against *C. parapsilosis* (FICI: 4.5) similar to that of planktonic cells. Regarding tyrosol and echinocandins, Kovács et al. (2017) described a remarkable decrease in caspofungin (1–16-fold) and micafungin (2–32-fold) MIC values in combination with tyrosol against *C. parapsilosis* sessile communities. Nevertheless, synergism was detected at 33% and 17% of *C. parapsilosis* isolates tested for caspofungin and micafungin, respectively (Kovács et al. 2017) (Fig. 2).

### Physiological and molecular background of antifungal effect exerted by tyrosol

Recently, several studies were published focusing on unravelling the background of tyrosol-related antifungal effects. In *C. parapsilosis*, exposure to 15 mM tyrosol resulted in a significantly increased production of reactive oxygen species compared to untreated control cells. This response was associated with increased activities of superoxide dismutase, glutathione peroxidase, and catalase in the tyrosol-treated cells, highlighting tyrosol’s role in inducing oxidative stress (Jakab et al. 2019) (Fig. 2). A similar pattern was observed in the case of *C. auris*, where the reactive oxygen species production was significantly elevated compared to untreated *Candida* cells, consistent with higher activities of superoxide dismutase, catalase, and glutathione peroxidase (Balla et al. 2023). Moreover, tyrosol treatment exerted a 25%, 37%, 34%, and 55% reduction in intracellular iron, manganese, zinc, and copper content in *C. auris*, further exacerbating the harmful effects of oxidative stress (Balla et al. 2023). The evaluation of total transcriptome changes of *C. parapsilosis* and *C. auris* revealed 261 and 142 up-regulated genes, as well as 181 and 108 down-regulated genes, respectively, in response to 15 mM tyrosol (Jakab et al. 2019; Balla et al. 2023). In *C. parapsilosis*, genes involved in ribosome biogenesis exhibited down-regulation; at the same time, genes associated with the oxidative stress response and ethanol fermentation were up-regulated. It is noteworthy that tyrosol exposure up-regulated several efflux pump genes, including *MDR1* and *CDR1*, as well as down-regulated the expression of the *FAD2* and *FAD3* genes. The results obtained align with the findings of Noskova et al. (2023), whose data suggest that tyrosol serves as a substrate for Mdr transporters. In *S. cerevisiae*, the tyrosol concentrations necessary to inhibit yeast growth and trigger *MDR* transporter induction were two orders of magnitude greater than the physiological concentration of tyrosol.

Interestingly, in the presence of supraphysiological concentration of tyrosol, metabolism was changed toward glycolysis and ethanol fermentation, while ergosterol biosynthesis genes were not affected, at least in *C. parapsilosis* (Jakab et al. 2019). Concerning metabolism, the indirect role of tyrosol was reported previously during glycolytic influx in *C. albicans* (Schwartz and Larsh 1982). The activities of hexokinase enzymes were doubled during the development of filaments as compared to the yeast form, indicating the higher glycolytic activity. Similar activity increases were detected in the case of phosphofructokinase, glucose-6-phosphate isomerase, fructose–bisphosphate aldolase, phosphoglycerate kinase, and pyruvate kinase (Doedt et al. 2004; Yin et al. 2004; Sexton et al. 2007). As tyrosol had already been established as a quorum-sensing molecule essential for filamentous growth (Chen et al. 2004), it was therefore relevant for its involvement in the glycolytic flux. In *C. auris*, genes associated with iron, fatty acid metabolism, and nucleic acid synthesis were down-regulated, while those related to antioxidative defense mechanisms and adhesion were up-regulated (Balla et al. 2023).

**Fig. 3:**
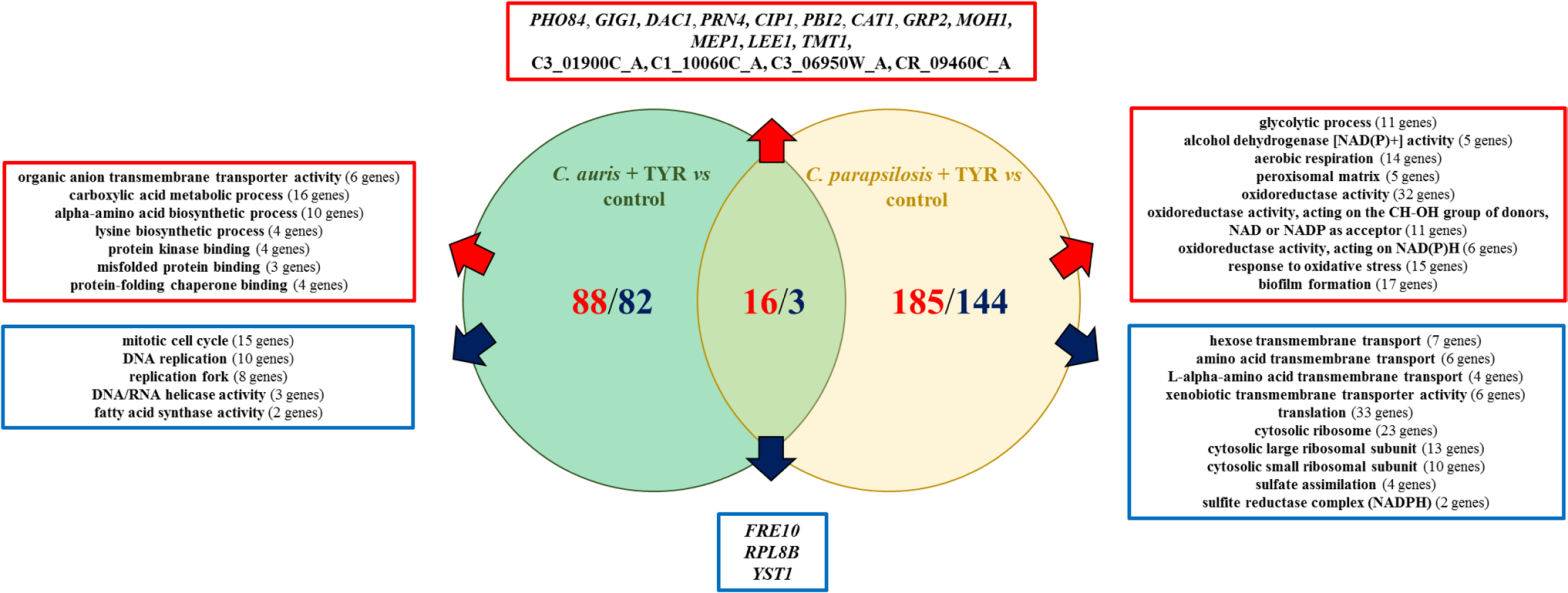
Summary of RNA-Seq data and gene enrichment analyses in case of tyrosol exposed *Candida auris* and *Candida parapsilosis* cultures. Orthologues of *Candida auris* and *Candida parapsilosis* genes were retrieved from the *Candida albicans* genome via the Candida Genome Database. In total, 155 genes were identified for which no orthologues could be detected in *Candida albicans*. Red boxes highlight enriched categories among upregulated genes/gene ontology terms (e.g., metabolic processes, amino acid biosynthesis, oxidative stress responses), while blue boxes indicate categories enriched among downregulated genes/gene ontology terms (e.g., membrane transport, ribosomal proteins, nucleic acid synthesis)

For *C. parapsilosis* and *C. auris*, total transcriptome analyses following tyrosol exposure have been reported by Jakab et al. (2019) and Balla et al. (2023) as we described above. Figure 3 summarizes the number of genes and gene ontologies significantly regulated upon tyrosol treatment compared to each other. Based on this comparison, *C. auris* displayed 88 up-regulated and 82 down-regulated genes, whereas *C. parapsilosis* showed 185 up-regulated and 144 down-regulated ones. Between the two species, sixteen genes were commonly up-regulated and three ones were commonly down-regulated. Genes down-regulated in both species include *FRE10*, *RPE1B*, and *YSP1*, whereas commonly up-regulated genes include *PHO84*, *GIG1*, *DAC1*, *PRN4*, *CIP1*, *PBI1*, *CAT1*, *GRP2*, *MOH1*, *MEP1*, *LEE1*, *TMT1*, *C3_01900C_A*, *C1_10060C_A*, *C3_06950W_A*, *CR_09460C_A* (Fig. 3).

Besides the abovementioned effects on yeasts exerted by tyrosol at supraphysiological concentrations, tyrosol exerts its effect directly or indirectly in overcoming stressed conditions in yeasts at physiological concentrations. Nath et al. (2021a, b) examined the relationship between metal toxicity and the effects of tyrosol. Metal toxicity is caused by induced oxidative stress, compromised DNA repair, inhibition of enzyme activity, and interruption of protein functions involved in cell proliferation, cell cycle progression, and apoptosis. Tyrosol has been shown to repair or enhance these functions in yeasts, suggesting that it likely plays a protective role in yeast cells exposed to metal stress under both natural and induced conditions, thereby supporting cell proliferation (Nath et al. 2021a, b). Nath et al. (2021a, b) recently demonstrated the protective role of tyrosol in yeast cells subjected to ethanol-induced oxidative stress. Their study showed that both *C. tropicalis* and *S. cerevisiae* were able to survive 12% ethanol stress when supplemented with 50 µM tyrosol. A comparable protective effect was previously observed by Cremer et al. (1999), who reported that tyrosol shielded *Candida* cells from reactive oxygen species generated by neutrophil granulocytes. Westwater et al. (2005) also reported that *C. albicans* cells in stationary phase were protected from oxidative stress in the presence of tyrosol.

### Potential uses of tyrosol in medicine

The majority of the current literature about the potential harnessing of tyrosol focuses on dental applications (Costa et al. 2021, 2025; Tang et al. 2023; Souza et al. 2024) (Fig. 2). The use of quorum-sensing molecules in oral hygiene products offers a novel alternative in dentistry for preventing oral infections caused by different *Candida* species. (Rocha et al. 2020; Wright and Ramachandra 2022). Prior to applying these compounds in oral hygiene for preventive purposes, further studies are needed to clarify their impact on the balance of the oral microbiome. Until recently, the number of studies dealing with the effects of quorum-sensing molecules on oral ecology was limited (Wright and Ramachandra 2022). It has been reported that tyrosol, when suspended in saliva at a concentration exceeding the solubility threshold, reduces the adherence of *C. albicans* and *C. glabrata* to acrylic resin. (Monteiro et al. 2015). do Vale et al. (2017) showed that tyrosol and chlorhexidine gluconate alone did not inhibit significantly the metabolism of the biofilm cells and number of CFU compared to the untreated control cells. The impact of this drug-drug combination on planktonic cells was characterized as antagonistic in the case of *C. albicans*, while it was deemed indifferent for *C. glabrata*. Moreover, the tested agents alone proved ineffective against the matured sessile cells. Nonetheless, the combined treatment exhibited a synergistic effect in reducing the number of hyphal forms developed by *C. albicans* at the incubation periods of 12 and 36 h for the drug combination. (do Vale et al. 2017). Previous studies have shown that these agents inhibited both early adhesion of *Candida* cells to acrylic surfaces (Monteiro et al. 2015) and biofilm forming ability by *Candida* species and *Streptococcus mutans* on acrylic resin and hydroxyapatite surfaces (Arias et al. 2016). These studies highlight the need for new oral strategies to combat diseases such as denture stomatitis and dental caries, which are caused by pathogenic biofilms formed by abovementioned species. Nevertheless, tyrosol showed no substantial reduction in acid production by *Candida* and *S. mutans*. (Monteiro et al. 2017a, b).

Among the alternative therapeutic approaches, the potential usage of nanoparticles related to quorum-sensing molecules can be highlighted, which have a higher ratio of surface area per volume reactivity than microparticles (Souza et al. 2019) (Fig. 2). Focusing on the various nanoparticles, including silver, gold and zinc, silver nanoparticles have been found to be the most effective (Samiei et al. 2016). Nanocomposites containing silver nanoparticles and calcium glycerophosphate show high antimicrobial and remineralising effects (Takamiya et al. 2021). Therefore, the combination-based usage of these nanocomposites with another compound (e.g., tyrosol) could increase the observed antimicrobial effect. Samiei et al. (2016) assessed their nanocompound in combination with tyrosol, which exhibited a synergistic effect against *C. albicans* and *S. mutans* with FICI values of 0.3 and 0.14. Yadav et al. (2022) developed tyrosol-functionalised chitosan gold nanoparticles to evaluate their antifungal and anti-biofilm effect against *C. albicans* and *C. glabrata*. They observed potent concentration-dependent fungicidal activity exerted by these nanoparticles; in addition, the majority of ergosterol synthesis-related genes were significantly down-regulated upon treatment (Yadav et al. 2022) (Fig. 2).

Among the various derivatives of tyrosol, the structural isomers 2-hydroxyphenethyl alcohol and 3-hydroxyphenethyl alcohol have been studied for their biological activities, with particular attention to their antioxidant and antimicrobial properties. Casadey et al. (2021) demonstrated that these two tyrosol isomers showed potent antioxidant and antimicrobial properties. Beyond antimicrobial activity, tyrosol and its derivatives have been reported to exhibit broader protective effects, including antioxidant and anti-inflammatory activities (Karković Marković et al. 2019; Guo et al. 2023). Moreover, tyrosol represents a key metabolic branch point within the phenylethanoid pathway, giving rise to other bioactive molecules such as salidroside (Guo et al. 2023). Certain tyrosol-1,2,3-triazole hybrids have shown moderate to good activities, indicating that they are a promising model for the synthesis of new acetylcholinesterase inhibitors (Bousada et al. 2020). In previous study, tyrosol was esterified on the primary hydroxyl group (Aissa et al. 2012). Synthetized compounds were assessed for their antimicrobial activity, and their antileishmanial effect. Based on these results, TyC _8_, TyC _10_ and TyC _12_ displayed good antibacterial and antileishmanial activities (Aissa et al. 2012). Zang et al. (2019) modified the alcohol hydroxyl groups of tyrosol. Their experimental results showed that most produced compounds exhibit 1,1-diphenyl-2-picrylhydrazyl and 2,2’-azino-bis(3-ethylbenzothiazoline-6-sulphonic acid) diammonium scavenging activities (Zang et al. 2019). These facts further underscore the relevance of tyrosol as a multifunctional compound with therapeutic potential.

### Industrial aspects of tyrosol

Tyrosol is a key ingredient in pharmaceuticals, nutraceuticals, and cosmetics, making it widely utilized in the pharmaceutical industry (Song et al. 2024) (Fig. 2). As a major pharmaceutical intermediate, it serves as a precursor to compounds like salidroside, icariside D2, and hydroxytyrosol. (Liao et al. 2018; Li et al 2018; Song et al. 2024). There are currently three industrial techniques for producing tyrosol. The first involves extracting it naturally from a range of plants, including olives. The second method is chemical synthesis, where toxic substances like phenylethyl alcohol and aromatic amines are used to produce tyrosol. The third method is biosynthesising tyrosol in different microorganisms from sugars (Guo et al. 2019).

In recent years, the number of studies focusing on biosynthetic aspects has been steadily increasing. Guo et al. (2019) introduced an exogenous pathway involved in catalysing tyrosine to tyrosol into *S. cerevisiae*. This modification resulted in 440 times higher tyrosol yield from the engineered strain compared with that of the control strain (126.7 ± 6.7 mg/g vs. 0.3 ± 0.01 mg/g dry cell weight). Song et al. (2024) reported that d-Erythrose 4-phosphate is a crucial precursor in the biosynthesis of tyrosol in *S. cerevisiae*. Therefore, the flux of d-Erythrose 4-phosphate directly influences the yield of tyrosol synthesis. To enhance the release of d-Erythrose 4-phosphate, a highly active phosphoketolase BA-C was utilized, resulting in a 151% increase in the total tyrosol yield (Song et al. 2024). Wang et al. (2025a, b) significantly improved the production of tyrosol by the overexpression of m^6^A modification writer Ime4 and reader Pho92, and the positive regulator Gcr2. In the case of the final tyrosol-producing engineered strain, the total yield of tyrosol was increased by 62-fold.

### Concluding remarks and future studies

In the past 20 years, tyrosol has been identified as one of the major quorum-sensing molecules in *Candida* species, which has a pivotal role in the regulation of biofilm formation, morphogenesis and virulence properties. Its potential virulence-quenching and interfering effects raised the attention of the scientific community to know about tyrosol as a potential tool in alternative antifungal strategies, disturbing quorum-sensing (Rodrigues CF and Černáková 2020; Kovács and Majoros 2020). Despite the great potential of tyrosol as an alternative antifungal treatment, several challenges remained unexplained. Despite current insights and increasing knowledge of tyrosol’s effects on *C. albicans* and different non-albicans species, the principles of the deep cellular molecular interactions involved remain largely unknown, which should be illuminated in the future. Furthermore, we know from the inter-kingdom interactions that these communications are often linked to quorum-sensing molecules, which significantly affect microbial growth, morphology, and virulence. Therefore, the effects of tyrosol on different bacterial species should be elucidated in the future to fully understand these tyrosol-based cross-species physiological and molecular interactions.

## Data Availability

No datasets were generated or analysed during the current study.
